# Investigating Factors of False-Positive Results of *Aspergillus* Galactomannan Assay: A Case–Control Study in Intensive Care Units

**DOI:** 10.3389/fphar.2021.747280

**Published:** 2021-12-20

**Authors:** Yu-Hsuan Hung, Hui-Hsiung Lai, Hui-Chuan Lin, Kuo-Shao Sun, Chung-Yu Chen

**Affiliations:** ^1^ Department of Pharmacy, Ditmanson Medical Foundation Chia-Yi Christian Hospital, Chiayi, Taiwan; ^2^ School of Pharmacy, Kaohsiung Medical University, Kaohsiung, Taiwan; ^3^ Division of Pulmonary and Critical Care Medicine, Department of Internal Medicine, St. Martin De Porres Hospital, Chiayi, Taiwan; ^4^ Chung-Jen Junior College of Nursing, Health Sciences and Management, Chiayi, Taiwan; ^5^ Department of Pharmacy, Kaohsiung Medical University Hospital, Kaohsiung, Taiwan; ^6^ Department of Medical Research, Kaohsiung Medical University Hospital, Kaohsiung, Taiwan; ^7^ Center for Big Data Research, Kaohsiung Medical University Hospital, Kaohsiung, Taiwan

**Keywords:** galactomannan, enzyme immunoassay, aspergillosis, intensive care unit, colistin

## Abstract

**Background:** Studies on false-positive galactomannan (GM) enzyme immunoassay (EIA) results and treatment for critically ill patients are scarce.

**Objectives:** The study aimed to determine the false-positive rate of GM-EIA and to probe the risk factors of false positivity among patients in the intensive care units (ICUs).

**Methods:** A case–control approach was conducted to review adult patients who had at least one GM-EIA result and were admitted to the ICU. Those who had no fungal culture were excluded. The clinical characteristics and critical care between patients with false-positive and true-negative GM index (GMI) were compared.

**Results:** Of 206 patients enrolled and with GM-EIA results, 20 (9.7%) were considered to have false-positive antigenemia, including 9 in bronchoalveolar lavages (BAL) and 11 in serum. A total of 148 (71.8%) were true-negatives. After paired grouping of 1:4, factors researched in the previous studies showed no significant difference. However, compared with the true-negatives, patients with positive GM test results but were incompatible with the diagnosis of invasive aspergillosis were more prone to the risk of false positivity due to the use of colistin inhalation. It seemed to be the only factor that significantly increased the risk of false positivity after multivariate analysis (adjusted odds ratio, 35.68; 95% CI, 3.77–337.51, *p* = 0.002).

**Conclusions:** Colistin inhalation treatment may contribute to false-positive GM-EIA results. The positive GMI among patients receiving colistin nebulization should be interpreted with caution.

## Introduction

Invasive aspergillosis (IA) is an important cause of fatality among immunocompromised patients. The incidence of IA has been increasing in Taiwan. An 11-year (2002–2012) follow-up report showed that 147 (36.12%) patients were admitted to the intensive care unit (ICU) (Sun et al., 2017). Not only neutropenic patients but also non-neutropenic patients are candidates to develop one or another form of aspergillosis (Vandewoude et al., 2016). The severity, multiple diseases, and high-dose steroids frequently used in the ICU may enhance the chance of aspergillosis (Meersseman et al., 2007). Early diagnosis of IA among critically ill patients became particularly important.

The Platelia *Aspergillus* assay for detecting circulating galactomannan (GM) has been widely used for diagnosing IA. GM is a component produced by the cell wall of *Aspergillus*. The convenience of sampling, high sensitivity, and shortened time of report make the test one of the useful auxiliary diagnostic tools for IA (Donnelly et al., 2020). But a variety of exposure factors have been reported to cause the false-positive results of this test, such as age, the existing disease, the infected bacteria, or the antibiotics, nutritional support, and even food taken during the hospitalization (Adam et al., 2004; Tomita et al., 2009; Ng et al., 2014; Avcu et al., 2017; Aigner et al., 2019). Samples from intensive care patients were frequently checked for GM in our institution. Under the complicated management for patients in the ICU, the results of the *Aspergillus* antigen test on critically ill patients should be more cautiously judged.

Due to the lack of research conducted on the ICU population receiving GM testing in Taiwan, this study aimed to determine the false-positive rate of GM enzyme immunoassay (GM-EIA) and to probe the risk factors of false positivity among this population.

## Materials and Methods

### Setting and Study Population

A case–control study was conducted at Ditmanson Medical Foundation Chia-Yi Christian Hospital in Taiwan, which provides both acute and chronic medical care services. GM testing was provided and conducted in the clinical laboratory of the study hospital. The study population comprised ICU hospitalized patients aged 20 and above who had at least one GM-EIA result from serum or bronchoalveolar lavage (BAL) between July 1, 2017, and October 31, 2019. Those without fungal cultures or who had been hospitalized for more than 30 days before the first assay were excluded from the analysis. The authors confirm that the ethical policies of the journal have been adhered to. The study was conducted according to the Declaration of Helsinki and was approved by the Institutional Review Boards of Ditmanson Medical Foundation Chia-Yi Christian Hospital (Research Ethics Committee No. IRB2019089), which waived the requirement for written informed consent.

### Data Collection and Definitions

Electronic medical records were used to obtain information, including baseline characteristics, comorbidities, concomitant medication, microbiological and histopathological reports, and laboratory data. Patients were followed up for 30 days after the test. All GM values of each patient during the study period were recorded. Diagnosis of the possibility of IA was based on revised definitions from the clinical algorithm (Bassetti and Bouza, 2017), referring to the AspICU (Blot et al., 2012) definition of the diagnosis of IA in the ICU (Supplementary Table S1). Patients were divided into 4 groups, according to host factors, clinical symptoms, and mycological evidence: proven IA, probable IA, colonization, and no IA. Patients with accurate pathology evidence were confirmed as proven IA; patients with appropriate host factors and clinico‐radiological signs and symptoms with a mycological criterion were classified as probable IA; patients with positive culture or positive antigen test were considered to have colonization if the three criteria were not all matched. Patients without positive results and also not all criteria matched were assumed as no IA.

The assay was considered positive when the GM index (GMI) reached ≥0.5 in serum and ≥0.7 in BAL. With the diagnosis of proven or probable IA, one positive GMI was defined as a true-positive result. One GMI on patients who had colonization or had no IA was considered to be false-positive. True-negative refers to colonization, and all GMIs were negative. Patients were considered to have false-negative results when all GMIs were negative but classified as proven IA or probable IA. The case group refers to patients with a false-positive value. The control group includes patients whose GMI was true-negative. For the control selection, four true-negative patients were randomly selected for each false-positive case by matching age and gender. The index date was the day when the first false-positive value or first true-negative test was examined.

### Statistical Analysis

This study used Pearson’s chi-squared test or Fisher’s exact test to analyze the distribution of categorical variables like diseases, medical treatments, and critical care between the two groups. Continuous variables as physiological test values were analyzed using Student’s t-test. Conditional regression was used to analyze the odds ratio of false positivity among exposure. Factors with at least borderline significance (*p* < 0.05) in the univariate analysis were subjected to a multivariate analysis by logistic regression analysis. All analyses were performed using SPSS for Windows 20.0.

## Results

### Patient Characteristics

During the research, a total of 308 patients who had been admitted to the ICU and tested for GM antigens were screened, including one readmission within 7 days. In total, 206 patients were enrolled (Supplementary Figure S1). Regarding the determination of the GMI, a total of 29 patients had a true-positive test value, 20 patients had a false-positive index, 148 patients had a true-negative test, and nine patients had a false-negative value. After pairing and grouping, the case group and the control group included 20 and 80 patients, respectively. Nine in BAL and 11 in serum were classified as false-positive GM.

The basic information, vital signs before the test, the severity of the condition, and the distribution of invasive treatment are shown in Table 1. The patients were mostly male, who account for up to 80% of the two groups. After the pairing, the average age was around 73–75 years. The acute physiology and chronic health evaluation II (APACHE II) of the two groups were the same-20 on average, which met the criteria for transferring to the ICU. There was no difference in the length of stay in the ICU. The coma scale of the two groups was roughly the same, with an average scale of 9 and 10. In terms of body temperature, the average was 35°C in the case group and 36°C in the control group. The mean arterial pressure (MAP) in the two groups was above 65 mmHg. In the part of biochemical items, the serum creatinine was both abnormal, averaged 1.8 and 1.9 mg/dl. The lactic acid of blood in the control group was 2.7 mmol/L, which was higher than that of the case group.

**TABLE 1:** Baseline admission characteristics of the false-positive and true-negative groups.

Parameter	Case (n = 20)	Control (n = 80)
Male (n, %)	17 (85.0%)	64 (80.0%)
Age (years)	75 (±14.2)	73 (±12.0)
Body mass index (kg/m^2^)	22 (±3.3)	23 (±4.7)
Glasgow Coma Scale	9 (±4.0)	10 (±3.9)
Temperature (°C)	35 (±4.6)	36 (±2.3)
Pulse (beats per minute)	91 (±28.2)	93 (±23.0)
Respiratory (times per minute)	20 (±9.4)	20 (±5.8)
Mean arterial pressure (mmHg)	83 (±18.7)	90 (±17.6)
APACHE II score	20 (±6.4)	20 (±7.4)
Length of ICU stay (days)	15 (±9.4)	16 (±15.7)
Serum creatinine (mg/dl)	1.8 (±3.1)	1.9 (±2.5)
Alanine transaminase (U/L)	37 (±31.8)	33 (±50.6)
Lactic acid of blood (mmol/L)	2.1 (±0.8)	2.7 (±1.8)
Septic shock	5 (25.0%)	13 (16.2%)
Aspiration pneumonia	4 (20.0%)	5 (6.2%)
Acute respiratory distress syndrome	1 (5.0%)	10 (12.5%)
Acute kidney injury	4 (20.0%)	19 (23.8%)
Solid tumor		
With chemotherapy	3 (15.0%)	11 (13.8%)
Without chemotherapy	1 (5.0%)	12 (15.0%)
Acute leukemia	0 (0.0%)	1 (1.2%)
Autoimmune disease	0 (0.0%)	3 (3.8%)

Note. ICU, intensive care unit; APACHE II, acute physiology and chronic health evaluation II.

Following the clinical algorithm (Table 2), the lung biopsy of the two groups did not show any presence of hyphae or tissue damage. In the host factor part, none of the eligible items were found in the case group. In the control group, 15 patients (18.8%) used an equivalent dose of prednisolone for more than 20 mg per day. Two patients had neutrophils of less than 500/mm^3^; 19 patients (23.7%) had chronic obstructive pulmonary disease (COPD) or bronchiectasis; 2 patients had liver failure up to Child-Pugh C; and 1 patient (1.3%) had severe complicated influenza. The proportion of abnormalities in the radiological evidence of the case group was greater than that of the control group. There were 10 patients (50.0%) in the case group with abnormal CT scan results and 33 patients (41.3%) in the control group; 15 patients (75.0%) in the case group had X-ray abnormalities and 44 patients (55.0%) in the control group. For mycological evidence, 4 patients (20.0%) in the case group had *Aspergillus* spp*.* versus 6 patients (7.5%) in the control group. The average GMI of the case group was 2.1, and the control group was 0.2.

**TABLE 2:** Diagnostic category of IA in the false-positive and true-negative groups.

Category	Case (n = 20)	Control (n = 80)
Pathology evaluation	0	0
Lung biopsy		
Host factors		
Prednisone ≥20 mg/day	0	15 (18.8%)
Neutrophils <500/mm^3^	0	2 (2.5%)
Chronic airway abnormality	0	19 (23.7%)
Decompensated cirrhosis	0	2 (2.5%)
T-cell immunosuppressants	0	1 (1.3%)
HSCT	0	1 (1.3%)
Organ transplantation	0	0
Human immunodeficiency virus	0	0
Severe influenza	0	1 (1.3%)
Clinical presentation^a^		
Signs on CT	10 (50.0%)	33 (41.3%)
Signs on X-ray	15 (75.0%)	44 (55.0%)
Mycological evidence		
Culture grew *Aspergillus* spp.	4 (20.0%)	6 (7.5%)
GM antigen ODI	2.1 (±2.0)	0.2 (±0.1)

Note. HSCT, hematopoietic stem cell transplantation; GM, galactomannan; ODI, optical density index.

a Signs including nodules, ground glass, halo sign, air crescent sign, consolidation, fungus ball, effusion, cavitation, and acute respiratory distress syndrome pattern.

### Factors Associated With Galactomannan Enzyme Immunoassay False Positivity

The fungi that may interfere with GMI found in the past literature were *Penicillium* spp., *Fusarium* spp., and *Candida glabrata*. *Mycelium* was mentioned, too. The results of culture in the control group were mostly *Candida* spp. There was no significant difference in false positivity between the two groups.

In terms of treatments in the ICU (Table 3), there was no difference in the association with the vasopressors, anesthetic, benzodiazepine, neuromuscular blocker, opioid, and false-positive results of the two groups. An injection containing soybean oil did not show a correlation either. As for the antibiotics, the use of penicillin and β-lactam antibiotic between the two groups showed no correlation with positive GMI. Among the patients, piperacillin/tazobactam was used by 12 patients (60.0%) in the case group and 52 patients (65.0%) in the control group, which did not reach a statistical difference. Cephalosporin and other antibiotics also did not increase the chance of false-positive results, except for colistin. The use of colistin showed a very significant correlation with false-positive results. From Table 4, the odds ratio was 33.86 (95% CI: 3.78–303.12, *p* = 0.002). After being corrected with MAP lower than 65 mmHg, APACHE II score greater than 15, and the use of morphine by using multivariate regression (forward stepwise regression model) analysis, the use of colistin had an odds ratio of 33.86 (95% CI: 3.78–303.12, *p* = 0.002), which showed a statistical difference.

**TABLE 3:** Relevance to the false-positive outcome between the treatment of the case and control groups.

Variables	Case (n = 20)	Control (n = 80)	Crude OR	95% CI	*p*
Vasopressor agents	12 (60.0%)	34 (42.5%)	2.03	0.748–5.508	0.165
Anesthetic	7 (35.0%)	35 (43.8%)	0.69	0.250–1.919	0.480
Benzodiazepine	18 (90.0%)	58 (72.5%)	3.41	0.731–15.941	0.118
Neuromuscular blocker	11 (55.0%)	33 (41.2%)	1.741	0.649–4.671	0.271
Opioid	12 (60.0%)	60 (75.0%)	0.50	0.1779–1.397	0.186
Soybean contained injection	7 (35.0%)	36 (45.0%)	0.658	0.238–1.823	0.421
Penicillin	13 (65.0%)	61 (76.2%)	0.578	0.202–1.658	0.308
β-Lactamase inhibitor	13 (65.0%)	62 (77.5%)	0.539	0.187–1.553	0.253
Amoxicillin/clavulanate acid	0 (0.0%)	8 (10.0%)	-		0.999
Ampicillin/sulbactam	2 (10.0%)	13 (16.2%)	0.573	0.118–2.772	0.488
Cefoperazone/sulbactam	1 (5.0%)	8 (10.0%)	0.474	0.056–4.024	0.494
Piperacillin/tazobactam	12 (60.0%)	52 (65.0%)	0.808	0.295–2.208	0.677
Cephalosporin	11 (55.0%)	44 (55.0%)	1.000	0.373–2.678	1.000
Carbapenem	8 (40.0%)	37 (46.2%)	0.775	0.286–2.099	0.616
Lincosamide	1 (5.0%)	1 (1.2%)	4.158	0.249–69.526	0.321
Glycopeptide	9 (45.0%)	37 (46.2%)	0.951	0.355–2.545	0.920
Aminoglycoside	2 (10.0%)	6 (7.5%)	1.370	0.255–7.361	0.713
Triazole	6 (30.0%)	21 (26.2%)	1.204	0.410–3.540	0.736
Metronidazole	1 (5.0%)	3 (3.8%)	1.351	0.133–13.721	0.799
Colistin	6 (30.0%)	1 (1.2%)	33.857	3.782–303.116	0.002^a^

a *p* < 0.05, achieved statistically significant difference.

**TABLE 4:** Multivariate analysis of potential factors affecting GM false positivity.

Variables	Crude OR	95% CI	Model 1		Model 2	
			AOR	95% CI	AOR	95% CI
MAP < 65 mmHg	4.53	0.84–24.41	4.25	0.65–27.67	33.86	3.78–303.12
APACHE II ≥ 15	1.65	0.43–6.25	1.20	0.27–5.39		
Colistin	33.86	3.78–303.12	26.73	2.83–252.67		
Morphine	3.86	1.16–12.85	2.65	0.64–10.94		

Note. MAP, mean arterial pressure; APACHE II, acute physiology and chronic health evaluation II; GM, galactomannan; AOR, adjusted odds ratio.

## Discussion

This retrospective study investigated factors associated with false-positive GM assay results in the ICU, and the results found a statistically significant association with treatment by colistin. Reviewing literature about false positivity of GM-EIA, none of them mentioned the correlation with the use of colistin, its prodrug, or its decomposition products. Colistin is indicated for acute or chronic infections due to sensitive strains of certain gram-negative bacilli. Ventilator-associated pneumonia in the ICU as a result of multidrug-resistant Gram-negative bacteria has contributed to the revival of the use of colistin. Considering the systemic adverse reactions caused by intravenous colistin, inhalation of colistin has become an alternative approach (Boisson et al., 2017). All of the patients, including 6 in the case group and 1 in the control, received colistin by inhalation in this study. The median and interquartile range (IQR) of the case group were 6.5 and 4.75 to 9.00, which are shown in Supplementary Tables S2, S3.

A study from Germany in 1999 may have indirect results related to our research. Bragon et al. investigated the factors in the prevalence of *Aspergillus* colonization in adults suffering from cystic fibrosis. They unexpectedly found that the colonization of *Aspergillus* in the patients was significantly related to the preventive antibiotics that the patients used. Of the patients colonized with *Aspergillus*, 65.1% had used prophylactic antibiotic inhalation treatment, which was tobramycin or colistin. And 44.3% of the patients under the same therapy did not develop *Aspergillus*. There was a proportional difference in the number of people receiving antibiotic inhalation therapy between the two groups (*p* = 0.035) (Bargon et al., 1999).

In 2010, Ben-Ami et al. (Ben-Ami et al., 2010) found that colistin has modest *in vitro* and *in vivo* fungicidal activities against *Mucorales* spp. and mycelia. Unexpectedly, they found that colistin had no *in vitro* activity against *Aspergillus fumigatus* and *Aspergillus terreus*. The selective activity suggested that the disruption of fungal membranes by colistin depends on the presence of specific receptors. This may explain why in patients with *Aspergillus* colonization colistin had no effects against *Aspergillus* spp. In patients colonized with *Aspergillus*, a positive optical density index (ODI) of 0.5–1.0 for GM in BAL may be seen without evidence of IA. The false positivity in our study may be attributed to the colonization of *Aspergillus* after treatment with colistin inhalation. Three patients in the case group used colistin and had *Aspergillus* in cultures.

Another possible reason may be the source of colistin manufacturing. Colistin (polymyxin E) is a polymyxin antibiotic produced by *Paenibacillus polymyxa* var. *colistinus*. One kind of bacterium, *P. polymyxa* A-8, was isolated from the soil sample under a pine tree located in the mountain region of Sichuan, China. The β-mannosidase gene from *P. polymyxa* A-8 was extracted (Bai et al., 2014). β-Mannosidase is one of the important mannan-degrading enzymes of mannan. GM is one of the subfamilies of mannan. With the possibility of β-mannosidase, in which its structure has similarities with GM, its existence during colistin extraction cannot be ruled out. All these remain a surmise.

Colistin was mentioned to have a positive result in another study (Francisco et al., 2006; Marty et al., 2006). (1,3)-β-d-Glucan (BG) is another antigen that can also be detected to diagnose IA by using a different assay. They found that colistin was tested positive for BG at reconstituted-vial concentrations, suggesting that the false-positive BG assays may occur when colistin is administered (Bai et al., 2014). This speculation cannot be applied to our result since no relevant literature shows that the presence of BG interaction with GM-EIA. But it is worth investigating. Furthermore, colistin was prescribed for treating bacterial infection; Supplementary Table S2 reports all bacterial species when patients use colistin. Despite that the bacteria could be responsible for a GM-like polysaccharide production rather than colistin, bacterial species did not have consistent characteristics, and it is hard to clearly identify the factors that may be related to the false-positive GM value.

To our knowledge, this is the first domestic article to investigate factors affecting the false-positive results of GM assay for emerging populations in the ICU of IA. Paired matching was used to equally distribute false-positive and true-negative results excluding true positive and doubtful situations (false GM but possible/probable IA) to the case and control groups as much as possible. In addition to the factors mentioned in the previous literature, we also include all other possible factors and surprisingly found a new discovery. Moreover, the diagnostic basis used in this study is widened compared with the guideline used in most of the research due to the different populations, in order to avoid excluding potential cases of *Aspergillus* infection. This study provides an applicable idea to consider in the diagnosis of *Aspergillus* infection.

There are several limitations in this study: first, when the test was positive, the physician would take clinical symptoms and host factors into consideration to determine the possibility of infection and prescribe antifungal agents for prevention or treatment. It will not be tested or will not undergo repeated inspections to verify the accuracy of this positive value due to antifungal agent exposure decreasing accuracy. Second, the method of retrieving medical records may be due to incomplete information or incomplete access to relevant information received by patients in other medical institutions, resulting in omissions in the data records, leading to deviations in the interpretation of test results. Third, the factors of food, rare disease, or environment mentioned in the previous literature had not been evaluated due to the absence of cases and difficulty of collecting data in this study. Fourth, despite GM testing having been performed on both BAL and serum in this study, there were different factors affecting BAL and serum specificity, which caused bias explaining the interpretation of GM in both sample types. Last, the small sample size also limits data analysis, making the difference in results impossible to show. Factors with different results from the previous literature cannot be further tested and verified by laboratory experiments due to limited resources and equipment.

## Conclusion

These real-world data (RWD) with one single-center ICU suggested that colistin inhalation treatment may contribute to false-positive GM-EIA results. The positive GMI among patients receiving colistin nebulization should be interpreted with caution. In the future, there should be laboratory analytical research to detect the components of different brands of colistin and the absorption ratio of the GM antigen of the related products after simulating colistin administered to humans through the inhalation route. Larger research with more persuasive evidence is necessary to explore the influence of colistin on GM false positivity.

## Data Availability

The original contributions presented in the study are included in the article/Supplementary Material. Further inquiries can be directed to the corresponding author.
