# Supplementary material for: Investigating Factors of False-Positive Results of Aspergillus Galactomannan Assay: A Case–Control Study in Intensive Care Units
Source: Front Pharmacol. 2021 Dec 20;12:747280. doi: 10.3389/fphar.2021.747280 (PMC8721279; doi:10.3389/fphar.2021.747280)
Supplement: Supplementary file 1 [file DataSheet1.PDF]

Investigating Factors of False-Positive Results of Aspergillus Galactomannan Assay: A Case-Control Study in Intensive Care Units

Supplemental file

eFigure 1 Inclusions and exclusions of study cohort

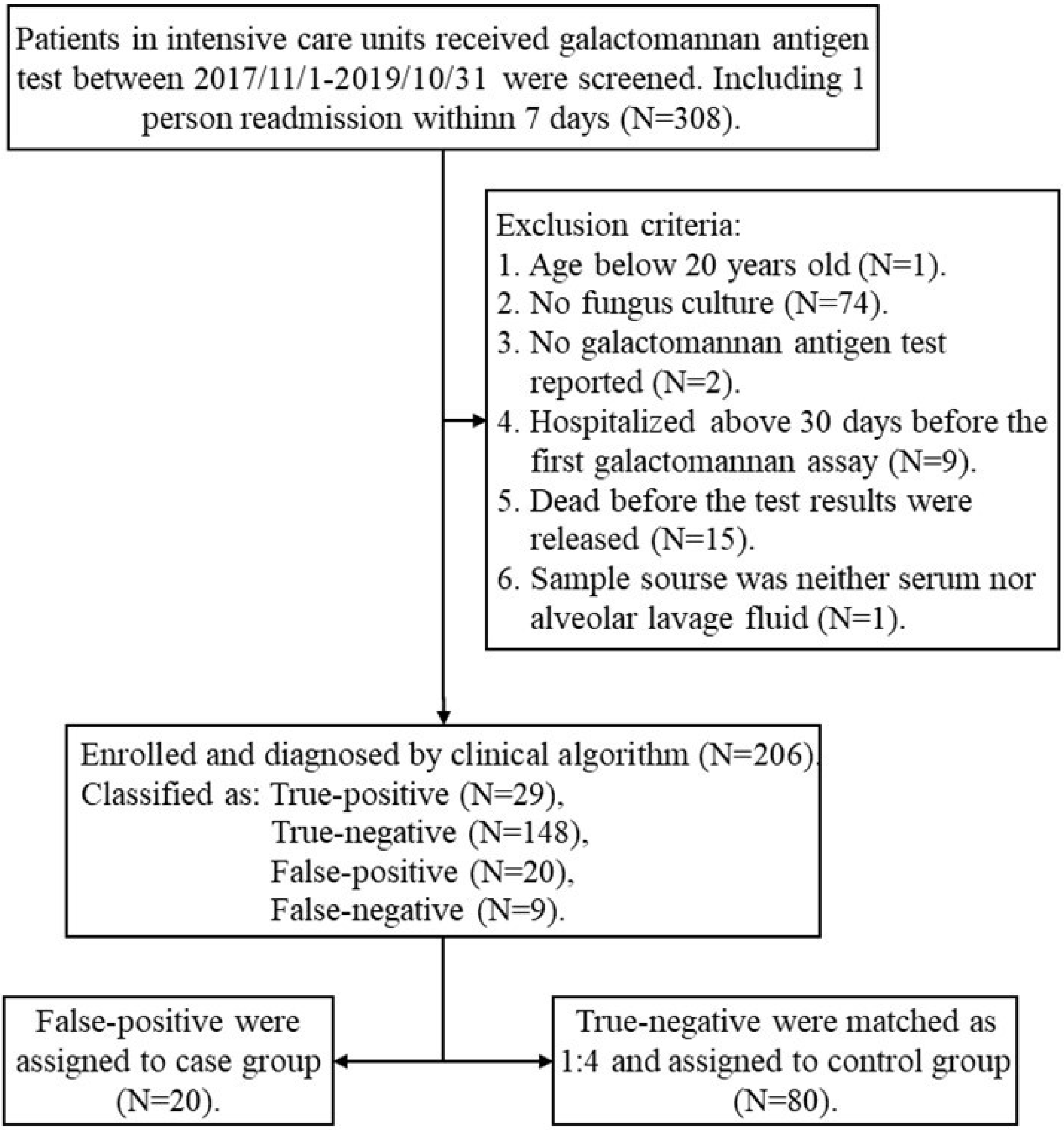

eFigure 1 Inclusions and exclusions of study cohort
