# Supplementary material for: Investigating Factors of False-Positive Results of Aspergillus Galactomannan Assay: A Case–Control Study in Intensive Care Units
Source: Front Pharmacol. 2021 Dec 20;12:747280. doi: 10.3389/fphar.2021.747280 (PMC8721279; doi:10.3389/fphar.2021.747280)
Supplement: Supplementary file 2 [file DataSheet2.docx]

**Investigating Factors of False-Positive Results of Aspergillus Galactomannan Assay: A Case-Control Study in Intensive Care Units**

eTable 1 Clinical algorithm for the diagnosis of IA† in non-neutropenic patients in the intensive care unit

eTable 2. Bacterial species before GM test in the seven patients treated with colistin.

eTable 3. Colistin usage in the seven patients.

eTable 1 Clinical algorithm for the diagnosis of IA† in non-neutropenic patients in the intensive care unit

| Category | Host factor | Clinical presentation | Mycological evidence |
| --- | --- | --- | --- |
| Proven IA | Not required | Not required | Pathology evaluation showing compatible hyphae and associated tissue damage and culture showing Aspergillus in specimen obtained by a sterile procedure from a normally sterile site. |
| Probable IA | At least one of the following:  Glucocorticosteroid treatment  Neutrophil abnormality  Chronic airway abnormality  Decompensated cirrhosis  Treatment with recognized  T-cell immunosuppressant  Haematological Malignancies/HSCT^‡^  Solid organ transplantation  Human immunodeficiency virus  Severe influenza | And clinical or radiological abnormalities consistent with a pulmonary infectious disease process that are otherwise unexplained. | At least one of the following non-definitive tests:  Cytology, direct microscopy and/or culture showing Aspergillus species in a lower respiratory tract specimen.  GM^§^ in serum ≥ 0.5 and/or in BAL^¶^ ≥ 0.7 |
| Colonization | Did not match all three criteria | | |
| Abbreviations: IA, invasive aspergiilus; HSCT, hematopoietic stem cell transplantation; GM, galactomannan; BAL, bronchoalveolar lavage. | | | |

| Patient | Sample source | Bacterial species | GM ODI | Sample sorurse |
| --- | --- | --- | --- | --- |
| Case A | Sputum culture (cough out)  Urine culture (catheter)  Sputum culture (endotracheal) | *Chryseobacterium indologenes*. *Staphylococcus aureus.*  *Corynebacterium* spp.  Coryneforms bacilli. | 3 | BAL |
| Case B | Blood culture  Sputum culture (cough out)  Urine culture | *Escherichia coli.*  G(-) bacilli4+, G(+) cocci in chains 3+, G(+) bacilli1+.  G(+) bacilli. | 3.45 | Blood |
| Case C | Blood culture  Sputum culture (endotracheal) | *Corynebacterium* spp.  no organism seen (WBCs:4+). | 1.29 | Blood |
| Case D | Urine culture (middle stream)  Sputum culture (cough out)  Aerobic culture (bronchial) | *Klebsiella pneumoniae*. *Proteus mirabilis*.  Mix upper respiratory tract microorganisms (WBCs:4+)  G(+) cocci (mix 2 type). Yeast-like (mix 2 type). | 0.74 | BAL |
| Case E | Urine culture (middle stream)  Sputum culture (cough out)  Sputum culture (endotracheal) | Yeast-like  Mix upper respiratory tract microorganisms  No growth after 48 hours (WBCs:4+) | 0.97 | BAL |
| Case F | Sputum culture (tracheotomy) | Mix upper respiratory tract microorganisms (WBCs:4+) | 0.87 | BAL |
| Control A | Urine culture (middle stream)  Blood culture  Aerobic culture (pus/Wound)  Anaerobic culture (pus/Wound)  Sputum culture (cough out)  Urine culture (catheter) | mix over 3 type  *Escherichia coli.*  *Escherichia coli*. *Enterococcus faecalis*.  *Veillonella* spp  Mix upper respiratory tract microorganisms  *Enterococcus faecium* | 0.23 | Blood |

eTable 2. Bacterial species before GM test in the seven patients treated with colistin.

| Patient | Quantity  (MU) | Frequency  (per day) | Duration  (day) | Period from the last dose (hour) | GM ODI | Sample sorurse |
| --- | --- | --- | --- | --- | --- | --- |
| Case A | 2 | twice | 12 | 9 | 3 | BAL |
| Case B | 4 | twice | 7 | 12 | 3.45 | Blood |
| Case C | 4 | twice | 6 | 0 | 1.29 | Blood |
| Case D | 4 | twice | 0.92 | 5.5 | 0.74 | BAL |
| Case E | 4 | twice | 8 | 2 | 0.97 | BAL |
| Case F | 4 | twice | 6 | 9.5 | 0.87 | BAL |
| Control A | 2 | twice | 0.29 | 4 | 0.23 | Blood |
| MU, million unit; GM, galactomannan; ODI, optical density index; BAL, bronchoalveolar lavage. | | | | | | |

eTable 3. Colistin usage in the seven patients.
